# Alternative management of diabetic ketoacidosis in a Brazilian pediatric emergency department

**DOI:** 10.1186/1758-5996-2-41

**Published:** 2010-06-16

**Authors:** Roberta D Savoldelli, Sylvia CL Farhat, Thais D Manna

**Affiliations:** 1 Pediatric Endocrine Unit, Instituto da Criança do Hospital das Clínicas da Faculdade de Medicina da Universidade de São Paulo, Brazil; 2 Emergency Unit, Instituto da Criança do Hospital das Clínicas da Faculdade de Medicina da Universidade de São Paulo, Brazil

## Abstract

DKA is a severe metabolic derangement characterized by dehydration, loss of electrolytes, hyperglycemia, hyperketonemia, acidosis and progressive loss of consciousness that results from severe insulin deficiency combined with the effects of increased levels of counterregulatory hormones (catecholamines, glucagon, cortisol, growth hormone). The biochemical criteria for diagnosis are: blood glucose > 200 mg/dl, venous pH <7.3 or bicarbonate <15 mEq/L, ketonemia >3 mmol/L and presence of ketonuria. A patient with DKA must be managed in an emergency ward by an experienced staff or in an intensive care unit (ICU), in order to provide an intensive monitoring of the vital and neurological signs, and of the patient's clinical and biochemical response to treatment. DKA treatment guidelines include: restoration of circulating volume and electrolyte replacement; correction of insulin deficiency aiming at the resolution of metabolic acidosis and ketosis; reduction of risk of cerebral edema; avoidance of other complications of therapy (hypoglycemia, hypokalemia, hyperkalemia, hyperchloremic acidosis); identification and treatment of precipitating events. In Brazil, there are few pediatric ICU beds in public hospitals, so an alternative protocol was designed to abbreviate the time on intravenous infusion lines in order to facilitate DKA management in general emergency wards. The main differences between this protocol and the international guidelines are: intravenous fluid will be stopped when oral fluids are well tolerated and total deficit will be replaced orally; if potassium analysis still indicate need for replacement, it will be given orally; subcutaneous rapid-acting insulin analog is administered at 0.15 U/kg dose every 2-3 hours until resolution of metabolic acidosis; approximately 12 hours after treatment initiation, intermediate-acting (NPH) insulin is initiated at the dose of 0.6-1 U/kg/day, and it will be lowered to 0.4-0.7 U/kg/day at discharge from hospital.

## Introduction

Diabetic ketoacidosis (DKA) is an acute and life-threatening complication of diabetes mellitus (DM) and consists of the biochemical triad of hyperglycemia, ketonemia, and metabolic acidosis [1]. Even in reference centers, 15 to 67% of diabetic patients still present DKA as the first manifestation of type 1 diabetes, mainly in children under 5 years old and in populations with difficult access to health services for socioeconomic reasons [2,3]. In Brazil, recent studies have shown that DKA was present in 32,8 to 41% of the patients at the time of diagnosis of type 1 DM [4-7], in agreement with international data. DKA is the most frequent cause of hospitalization and mortality among type 1 diabetes children and adolescents, and it accounts for approximately 50% of all deaths of DM individuals up to 24 years of age [8]. However, type 2 DM patients are also susceptible to DKA under stressful situations such as infections, surgery or trauma [9]. In developed countries, DKA mortality rate is 0.15% to 5% [10,11] and it is mainly due to cerebral edema, occurring in about 1% of the cases [2]; epidemiological data on DKA morbidity and mortality are still scarce in Brazil. An accurate diagnosis and careful management of the situation are crucial to reduce the related complications. In our country, there are few pediatric intensive care unit (ICU) beds in public hospitals, which are usually occupied by children who demand ventilatory support; therefore most of DKA patients will be managed in general emergency wards.

We report here a local experience with an alternative fluid and insulin therapy protocol designed to abbreviate the time on intravenous infusion line for the management of uncomplicated DKA cases in a Brazilian pediatric emergency department.

## DKA: pathophysiology, clinical condition and treatment

DKA is the consequence of a complex disturbance in carbohydrate, protein and lipid metabolism, due to relative or absolute insulin deficiency and elevated counterregulatory hormone (glucagon, catecholamines, cortisol and growth hormone) levels [1,12], causing accelerated catabolism with increased glucose production by the liver (via glycogenolysis and glyconeogenesis) and decreased peripheral glucose uptake, which creates progressive hyperglycemia.

Blood glucose levels higher than 180 mg/dl usually surpass renal glucose absorption threshold, leading to glycosuria and osmotic diuresis, which causes water and electrolytes (sodium, potassium, phosphorus and magnesium) losses, determining a severe depletion in intravascular volume that may impair kidney perfusion and further compromise the renal ability to excrete glucose worsening hyperglycemia [1,2], and consequently the serum hyperosmolality [13]. However, in DKA the clinical assessment of dehydration is easily underestimated, as such clinical signs are determined mainly by the extracellular volume, which is maintained by hyperosmolality.

Extreme insulinopenia associated with accelerated catabolism generates a high degree of lipolysis and a consequent increase in hepatic oxidation of free fatty acids into ketone bodies (beta-hidroxybutirate and acetoacetate), which are highly responsible for DKA metabolic acidosis [12,14]. Also, the hypovolemia that results from osmotic diuresis and vomiting decreases peripheral perfusion, leading to the accumulation of lactic acid in the tissues, which contributes to metabolic acidosis on a lower degree. Generally, acetoacetate is converted into acetone, which is eliminated by urine and by breathing; however, lactic acidemia present in DKA increases acetoacetate conversion into beta-hidroxybutirate, which impairs the renal ability to excrete such ketoacids, characterizing DKA as an increased anion gap metabolic acidosis [15] (Table 1).

**Table 1:** Biochemical criteria for DKA definition and categorization [12,16]

	DKA		
	
	mild	moderate	severe
**Blood glucose (mg/dl)**	> 200	>200	>200

**Serum pH**	< 7.3	< 7.2	< 7.1

**Serum bicarbonate (mEq/L)**	< 15	< 10	< 5

**Ketonemia (mmol/L)**	> 3	> 3	> 3

**Ketonuria**	present	present	present

**Serum osmolality (mOsm/kg)**	variable	variable	variable

**Anion gap (normal = 12 ± 2 mEq/L)**	> 12	>12	>12

Hypertonicity provokes an osmotic flux of water from the intracellular to the extracellular compartment and nearly all body cells (except for neurones) go through an intracellular dehydration process. This water transcellular shift also generates an extracellular efflux of potassium, further aggravated by metabolic acidosis, causing a high intracellular potassium deficit [12,15,16]. The total-body potassium deficit occurs mainly due to vomiting and osmotic diuresis; however, despite the total depletion, serum potassium levels at diagnosis may be increased, normal or decreased [12,15].

Hyperglycemia is the determining factor of serum hyperosmolality, responsible for the osmotic flux of water to extracellular space, which may cause dilutional hyponatremia; it is estimated that for each 100 mg/dl blood glucose concentration above the limit of 100 mg/dl, there is a 1.6 mEq/L reduction in serum sodium. In general, there is also a depletion of total-body phosphate related to osmotic diuresis too, and an accentuated fall in its serum levels is observed after starting insulin replacement, which provokes a rapid intracellular shift of phosphate [12,17].

**Figure 1:**
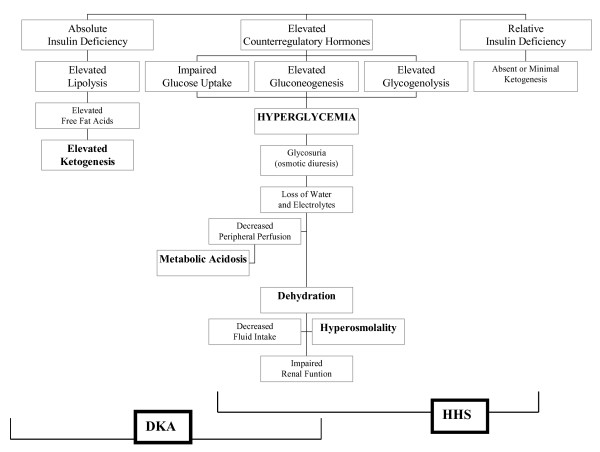
**Pathophysiology of diabetic ketoacidosis (DKA) and of hyperglycemic hyperosmolar state (HHS) (modified from **[16]).

Dehydration, hyperosmolality, acidosis and electrolytic disorders lead to the release of counterregulatory hormones, perpetuating the decompensation cycle [18] (Figure 1).

In severe cases of DKA, hyperpnea may cause a very low pCO2 and, consequently, there will be a decreased CO2 diffusion through the hematoencephalic barrier determining a low pCO2 in the smooth muscles of cerebral vessels and an increased extracellular fluid pH, which can provoke cerebral vasoconstriction [19]. The biochemical criteria for DKA definition and categorization can be found in Table 1[12,16].

Intercurrent illnesses in addition to intense physical or emotional stress are risk factors for DKA; however, difficulties in coping with insulin and diet therapies, peripubertal emotional instability, poor metabolic control, lack of family support, psychiatry disorders, limited access to medical services, insulin pump failure, use of drugs with hyperglycemiant effect may increase the risk for DKA [5,14,16].

The leading clinical manifestation is dehydration, which may be mild, moderate or severe with hemodynamic instability (capillary refill time > 3 seconds, weak pulses, cool extremities, tachycardia, normal or lower blood pressure) and despite dehydration, with only a few exceptions, urine output is considerable. Intense ketogenesis causes a rampant clinical condition, with nausea, vomiting, abdominal pain, hyperpnea (from mild to Kussmaul breathing), facial redness and ketotic breath.

Initial assessment of the DKA patient must include detailed physical examination, focusing on the circulatory state (pulse, blood pressure and peripheral perfusion), dehydration degree, level of consciousness, presence of acidotic breathing, ketotic breath, or signs of associated infectious conditions. Finger-stick capillary blood glucose and ketonemia testing must be conducted at the bedside, ketonuria and glycosuria shall be monitored using urine reactive strips. Biochemical assessment will include venous blood glucose, blood gases, serum electrolytes (sodium, potassium, chloride, phosphorus), urea and creatinine, allowing for the calculation of serum osmolality and anion gap [12,20] (formulas in Table 2).

**Table 2:** Formulas used to calculate serum osmolality and anion gap in DKA [12]

Effective osmolality (normal = 280-290 mOsm/kg)	=	2 × [Na^+ ^+ K^+^] + glucose (mg/dl)/18
**Anion gap (normal = 12 ± 2 mEq/L)**	=	Na^+ ^(mEq/L) - [Cl^- ^+HCO_3_^-^(mEq/L)]

In severely obtunded, unconscious patients or with signs of respiratory failure, orotracheal intubation and nasogastric tube should be placed in order to establish a safe air flow [12,21]. In this situation, it is recommended to preserve "adaptive tachypnea" for the acidosis level [22,23], in order to avoid exaggerated vasoconstriction due to an accentuated drop in pCO2 levels, or otherwise increased vasodilatation secondary to the excessively increased pCO2 levels.

Moderate-to-severe dehydrated patients should be managed in a medical center specialized in critical care, where they can be constantly monitored and reassessed [14,20].

DKA treatment aims to correct dehydration, electrolytic disorders, hyperglycemia and acid-base imbalance, as well as identifying and controlling the triggering factors in order to avoid the complications resulting from such procedures [12,14,20].

Replacement of body fluids is important for repair of circulation and renal functions, enhancing glucose excretion and decreasing levels of counterregulatory hormones that stimulate hyperglycemia [24].

**Figure 2:**
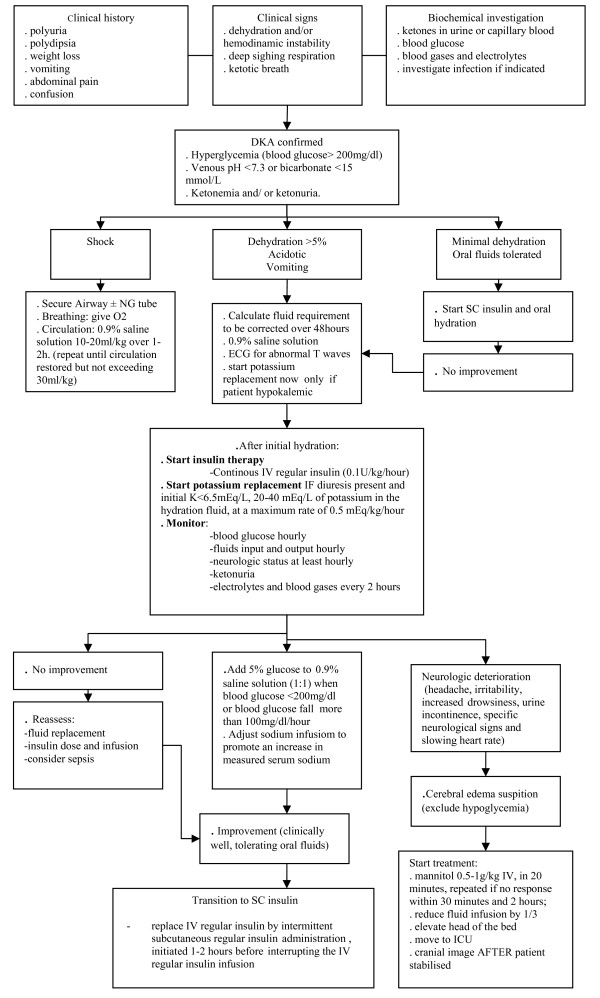
**Management of diabetic ketoacidosis (adapted from **[20]).

The objectives of fluid and electrolyte replacement therapy in DKA are restoration of circulating volume, replacement of sodium and the extracellular and intracellular fluid deficit of water, restoration of glomerular filtration with enhanced clearance of glucose and ketones from the blood, and avoidance of excessive rates of fluid administration so as not to exacerbate the risk of cerebral edema [14,20] (Figure 2).

Initial intravenous fluid administration should begin immediately with an isotonic solution (0.9% sodium chloride) in a 10 to 20 ml/kg bolus over 1 to 2 hours. Subsequent fluid management (deficit replacement) should be with 0.9% normal saline for at least 4-6 hours; thereafter, deficit replacement should be with a solution with a tonicity greater than or equal to 0.45% saline with added potassium [14,20,25]. The remainder replacement intravenous fluid should be given over at least 48 hours. Urinary losses should not be added to the calculation of replacement fluids [14,20,25].

Potassium replacement may be initiated as soon as urine output is documented, as long as serum potassium levels are ≤ 6.5 mEq/L, usually from the second hour of resuscitation phase, and 20-40 mEq/L of potassium will be included to the fluid regimen, at a maximum rate of 0.5 mEq/kg/hour [12,14,26]. There is no consensus about administering potassium chloride or potassium phosphate, and a total correction with a 19.1% potassium chloride (KCl) solution is also accepted. If potassium phosphate salts are used, it is recommended to use half of 25% monopotassium phosphate (1 ml = 1.8 mEq of potassium) and half of 19.1% KCl (1 ml = 2.5 mEq of potassium) [14,20]

Phosphate levels are usually low in DKA and may drop even further after insulin therapy is initiated; there is a theoretical reason for its replacement that is the decrease in tissue oxygenation due to a fall in 2,3 diphosphoglycerate (2,3 DPG) levels (phosphate depletion syndrome), however, the benefits of its replacement have not yet been proven, since it may induce hypocalcemia and hypomagnesemia [26,27].

Insulin therapy must be initiated 1-2 hours after the beginning of fluid resuscitation, if there has already been an improvement in peripheral perfusion; then, low insulin doses may be administered by intravenous (IV), intramuscular (IM) or subcutaneous (SC) route [1,18,28,29]. Continuous IV infusion of low doses of human regular insulin has been chosen most often as it causes more predictable fall of blood glucose levels, and it allows for more immediate dose adjustments [12,18,20]. Nevertheless, IV insulin administration requires another venous line to allow independent handling of fluid hydration and of the insulin infusion rate.

Human regular insulin should be infused at the dose of 0.1 U/Kg/hour (for instance: 50 U in 500 ml of 0.9% saline solution, where 1 ml = 0.1 U) aiming to promote a plasma glucose concentration fall at a rate of 50-90 mg/dl/hour [12,14,20]. Capillary blood glucose should be measured hourly, as well as the rate of falling of plasma glucose concentrations; if the patient demonstrates marked sensitivity to insulin, 5% glucose shall be added to the saline solution or insulin dose may be reduced to 0.05 U/Kg/hour, so that IV insulin therapy is kept until ketosis and metabolic acidosis are under control [12,14,20].

In most DKA events, metabolic acidosis control will occur as a consequence of fluid replacement and insulin therapy; however, in severe cases when pH < 6.9, alkali therapy may be indicated in order to prevent decreased cardiac contractility and peripheral vasodilatation that could further impair tissue perfusion [20]. Bicarbonate administration of 1-2 mEq/kg would be done over 1-2 hours or according to the following formula:

Bicarbonate to be administered (mEq) = (15 - Bicarbonate found) × 0.3 × weight (kg)

Venous blood sampling should be obtained every 2 hours for monitoring plasma glucose, serum electrolytes (sodium, potassium, chloride, phosphorus), blood urea, creatinine levels, and blood gases until metabolic acidosis is corrected. In case a phosphate solution is used at rehydration phase, it is also recommended to monitor calcium and magnesium levels [12,14,26].

When metabolic acidosis is controlled (pH > 7.3, bicarbonate > 15, and/or anion gap = 12 ± 2) [12,14,20], IV regular insulin may be replaced by intermittent SC regular insulin administration (0.1 U/kg every 4 hours), which must be initiated 1-2 hours before interrupting the IV regular insulin infusion.

## Alternative DKA treatment protocol

Our alternative treatment protocol for uncomplicated DKA cases was designed to abbreviate the time on intravenous fluid and insulin replacement in order to simplify the management of this condition in the general pediatric emergency wards. The objectives of this alternative protocol are: a) restoration of extracellular fluid volume; b) replacement of sodium and potassium deficits; c) control of metabolic acidosis and ketonemia; d) control of hyperglycemia; e) reduction of risk of cerebral edema.

In this protocol, fluid replacement is initiated before insulin therapy, as normalization of peripheral perfusion is also necessary for insulin to reach the receptors in the target tissues. The aim of fluid therapy is expanding the intravascular compartment until peripheral circulation is normalized; it is initiated with a 0.9% sodium chloride solution (normal saline) - 20 ml/kg/hour (maximum 1.000 ml/hour) and additional parts of normal saline - 10 to 20 ml/kg/hour will be repeated, if necessary, until restoration of peripheral perfusion usually after 4-6 hours; in the presence of shock, 50 ml/kg may be infused over the first hour (maximum 1.000 mL/hour). During resuscitation phase, if blood glucose levels reach 200 mg/dl or lower, a 5% glucose solution may be added to 0.9% saline solution (half and half).

Maintenance fluid (100 ml/100 kcal/day) will be administered only if oral fluids could not be tolerated; therefore, total fluid deficit will not be replaced by intravenous fluids. Gradual and continuous oral rehydration will promote a slower correction of hyperosmolality.

Since 1996, we use in our emergency department an alternative insulin therapy protocol with SC rapid-acting analog (lispro or aspart), which was validated by a randomized clinical trial comparing 30 DKA episodes treated in such protocol with other 30 episodes treated with continuous intravenous insulin infusion [30]. In this regard, a recent evidence-based review concluded that the use of subcutaneously administered rapid-acting insulin analogs is safe and effective for the management of uncomplicated DKA [31]. All the 60 DKA episodes enrolled in our randomized clinical trial received the same fluid replacement therapy described above. The biochemical characteristics of both branches of the study during the first 30 hours of DKA management are illustrated in Table 3.

**Table 3:** Biochemical characteristics (Mean ± SD) of DKA episodes treated with subcutaneous rapid-acting insulin analog (SC Analog; n = 30) and continuous intravenous regular insulin (IV Insulin; n = 30) during the first 30 hours of management with the same alternative fluid replacement therapy

Time	Insulin Therapy	Blood Glucose (mg/dl)	pH	**HCO**^-^**_3 _(mmol/L)**	β-OH butyrate (mmol/L)	**Na**^+ ^**(mEq/L)**	**Cl(mEq/L)**	**K**^+ ^**(mEq/L)**	Phosphorus (mg/dl)	Urea (mg/dl)	Serum Osmolality (mOsm/kg)
**Admission**	IV Insulin	442.7 ± 146.2	7.18 ± 0.1	9.08 ± 3.61	8.45 ± 2.75	136.7 ± 4.1	105.1 ± 9.4	5.1 ± 0.8	5.1 ± 1.6	37.8 ± 15.4	312.9 ± 13.8
	SC Analog	443.7 ± 143.2	7.17 ± 0.1	10.38 ± 4.74	8.16 ± 3.53	137.1 ± 4.8	109.9 ± 6.6	5.1 ± 0.6	5.0 ± 1.3	37.5 ± 10.1	311.9 ± 13.7
											
**Hour 2**	IV Insulin	332.9 ± 74.6	7.19 ± 0.11	9.76 ± 4.08	7.08 ± 2.56	139.8 ± 4.5	108.3 ± 9.9	4.8 ± 0.8	3.9 ± 1.0	34.1 ± 14.3	310.9 ± 12.4
	SC Analog	335.7 ± 126.8	7.15 ± 0.13	9.83 ± 5.71	7.54 ± 3.53	139.0 ± 5.6	107.9 ± 8.4	5.0 ± 0.9	4.3 ± 1.1	32.5 ± 10.0	305.8 ± 16.2
											
**Hour 6**	IV Insulin	222.6 ± 42.1	7.24 ± 0.12	9.76 ± 4.08	4.08 ± 2.58	140.1 ± 5.4	109.8 ± 9.3	4.4 ± 0.9	3.3 ± 1.2	30.7 ± 13.0	305.4 ± 13.7
	SC Analog	213.6 ± 66.8	7.22 ± 0.12	9.83 ± 5.71	5.18 ± 3.17	140.4 ± 5.4	112.4 ± 9.9	4.6 ± 0.8	3.3 ± 0.9	28.6 ± 8.6	302.2 ± 13.3
											
**Hour 12**	IV Insulin	187.7 ± 86.3	7.31 ± 0.09	15.79 ± 4.42	2.61 ± 2.18	139.1 ± 4.0	110.2 ± 6.9	4.1 ± 0.6	2.8 ± 0.8	24.8 ± 12.9	297.6 ± 11.2
	SC Analog	241.4 ± 82.7	7.29 ± 0.08	14.25 ± 6.10	3.29 ± 2.84	137.5 ± 3.9	108.3 ± 6.9	4.1 ± 0.5	3.0 ± 1.0	27.0 ± 7.3	296.5 ± 10.34
											
**Hour 18**	IV Insulin	241.9 ± 122.0	7.36 ± 0.04	18.39 ± 3.66	1.57 ± 1.82	137.8 ± 4.0	104.8 ± 8.1	3.9 ± 0.4	2.6 ± 0.8	26.9 ± 12.0	297.6 ± 11.2
	SC Analog	210.6 ± 100.7	7.31 ± 0.05	16.78 ± 6.27	2.0 ± 2.0	138.1 ± 2.8	109.0 ± 6.3	3.8 ± 0.5	3.0 ± 1.0	27.2 ± 8.0	296.5 ± 10.3
											
**Hour 30**	IV Insulin	269.7 ± 133.5	7.37 ± 0.07	19.52 ± 5.10	0.84 ± 0.91	138.3 ± 5.2	106.9 ± 6.8	3.7 ± 0.5	2.5 ± 1.1	26.2 ± 10.8	302.4 ± 10.1
	SC Analog	245.9 ± 91.1	7.37 ± 0.06	18.19 ± 4.72	1.12 ± 1.59	137.2 ± 3.0	106.8 ± 7.9	4.0 ± 0.4	2.5 ± 0.8	26.5 ± 8.3	297.2 ± 5.4

According to this alternative insulin therapy protocol, SC rapid-acting analog is initiated at a dose of 0.15 U/kg every 2 hours, reducing it to 0.1 U/kg in case the rate of falling of plasma glucose concentrations exceeds 100 mg/dl/hour, until resolution of metabolic acidosis [30,32]. In order to avoid hypoglycemia, 5% glucose solution will be added to saline solution (1:1) when blood glucose levels are close to 200 mg/dl. Approximately 12 hours after the treatment beginning, when metabolic acidosis is usually under control, intermediate-acting (NPH) insulin is initiated at the dose of 0.6-1 U/kg/day. This higher dose is required to compensate for the insulin resistance determined by DKA, and it will be frequently associated with extra SC 0.1 U/kg doses of rapid-acting analog according to capillary blood glucose monitoring every 3 hours. NPH insulin dose will be lowered to 0.4-0.7 U/kg/day at discharge from hospital.

Oral fluids may be introduced upon improvement of the level of consciousness, nauseas and vomiting. If oral acceptance is not enough to keep blood glucose levels between 90 and 180 mg/dl [33-36], an IV fluid solution with 5% glucose will be maintained, so that insulin therapy is not interrupted before the ketogenic process is totally under control. Oral potassium replacement may be maintained based on serum potassium measurements [20].

## DKA-related complications

Patients must be monitored for possible complications, such as hypoglycemia, hypokalemia, cardiac arrhythmia, venous and arterial thrombotic phenomena, and more rarely pneumomediastinum, pneumopericardium and subcutaneous emphysema [37,38], however, cerebral edema is the main cause of DKA-associated mortality [12,14].

Cerebral edema may occur in 0.5-1% of all DKA cases in childhood, and usually develops 5-15 hours after treatment has started, followed by high morbidity rates, with permanent neurological handicap from 10-25% and mortality from 20-25% [12]. Risk factors associated with cerebral edema would be: age inferior to 5, recent DM diagnosis, long-standing symptoms, hypocapnia, severe acidosis, bicarbonate treatment for acidosis, excessive volume replacement in the first 4 hours and insulin administration in the first hour of fluid replacement [12]. However, some studies suggested that cerebral edema occurs most frequently and with greatest severity in children with greater dehydration and hypocapnia [23,39-41]. In a recent review, Glaser pointed to the possible involvement of cerebral ischemia and reperfusion injury in causing DKA-related cerebral edema and cerebral injury, suggesting a need to re-examine DKA treatment protocols to determine whether injury could be lessened by optimizing rehydration strategies [42].

Cerebral edema diagnosis is clinical and the presence of the following symptoms shall alert to its occurrence: headache and slowing of heart rate, change in neurologic status (irritability, increased drowsiness, urine incontinence), specific neurological signs such as cranial nerve palsies, decerebration or decortication posture [12]. In case of clinical suspicion, treatment must be started quickly by administering mannitol 0.5-1 g/kg IV, in 20 minutes, which may be repeated if there is no response within 30 minutes and 2 hours; fluid infusion must be reduced by 1/3, and the head of the bed should be elevated. In case of suspicion of herniation, the patient must also be intubated in order to maintain hyperventilation (pCO_2 _between 27-30 mmHg); however, aggressive hyperventilation, with pCO_2 _< 22 mmHg, must be avoided because it has been associated with poor outcome [12,14]. After the treatment is initiated, a cranial tomography will be conducted in order to exclude cerebral thrombosis or hemorrhage [12].

Since this alternative DKA management protocol was adopted in our emergency department in 1996, approximately 30 DKA episodes per year have been treated, and neither cerebral edema nor related deaths were reported in this period [30]. The use of elevated volumes of saline solution (0.9% sodium chloride) could be associated with development of hyperchloremic acidosis [43], however such complication has not been demonstrated during the 30 hours of treatment (Table 3). On the other hand, a positive tendency of natremia during rehydration has been associated with a favorable outcome and less incidence of brain edema [39,44].

An important point of care in DKA treatment is preventing its recurrence, since all of its causes are preventable. Insulin omission or difficulties to deal with stressful events of life are the most common causes. Therefore, patients and family members must be educated and undergo training for intensive monitoring, ketone detection and insulin dose adjustments during intercurrent illness periods. Adult supervision of insulin administration, psychological assessment and support for patients and their family may contribute to the reduction in frequency of recurrent DKA [8,12].

## Conclusion

We presented here a local experience with an alternative protocol designed to abbreviate the time on intravenous infusion lines in order to facilitate DKA management in general emergency wards. The main differences between this protocol and the international guidelines are: intravenous fluid will be stopped in the moment when oral fluid is well tolerated and total deficit will be replaced orally; if electrolyte measurements still indicate need for replacement, it will be given orally; SC rapid-acting insulin analog is administered at 0.15 U/kg dose every 2-3 hours until resolution of metabolic acidosis; approximately 12 hours after treatment beginning, intermediate-acting (NPH) insulin is initiated at the dose of 0.6-1.0 U/kg/day, and this dose will be lowered to 0.4-0.7 U/kg/day at discharge from hospital. Since 1996, when this alternative DKA management protocol was adopted in our emergency department, neither cerebral edema nor related deaths were reported.

## List of abbreviations

DKA: diabetic ketoacidosis; DM: diabetes mellitus; NPH: neutral protamine Hagedorn; U: units; BE: base excess; Na^+^: sodium; K^+^: potassium; CO_2_: carbon dioxide; pCO_2_: partial pressure of carbon dioxide; HCO_3_^-^: bicarbonate; β-OH: beta-hydroxy; HHS: hyperglycemic hyperosmolar state; KCL: potassium chloride; 2,3 DPG: 2,3 diphosphoglycerate; IV: intravenous; SC: subcutaneous.

## Competing interests

The authors declare that they have no competing interests.

## Authors' contributions

RDS participated in acquisition of data and helped to draft the manuscript. SCLF participated in the design of the study and in acquisition of data. TDM conceived of the study, participated in its design and coordination and helped to draft the manuscript. All authors read and approved the final manuscript.
